# Optimization of Johnson–Cook Constitutive Model Parameters Using the Nesterov Gradient-Descent Method

**DOI:** 10.3390/ma16155452

**Published:** 2023-08-03

**Authors:** Sergey A. Zelepugin, Roman O. Cherepanov, Nadezhda V. Pakhnutova

**Affiliations:** Tomsk Scientific Center of the Siberian Branch of the Russian Academy of Sciences, 634055 Tomsk, Russia; szel@yandex.ru (S.A.Z.); nadin_04@mail.ru (N.V.P.)

**Keywords:** Johnson–Cook (JC) constitutive model, Taylor impact test, high strain-rates, shock waves, finite element simulation, Nesterov gradient-descent method

## Abstract

Numerical simulation of impact and shock-wave interactions of deformable solids is an urgent problem. The key to the adequacy and accuracy of simulation is the material model that links the yield strength with accumulated plastic strain, strain rate, and temperature. A material model often used in engineering applications is the empirical Johnson–Cook (JC) model. However, an increase in the impact velocity complicates the choice of the model constants to reach agreement between numerical and experimental data. This paper presents a method for the selection of the JC model constants using an optimization algorithm based on the Nesterov gradient-descent method. A solution quality function is proposed to estimate the deviation of calculations from experimental data and to determine the optimum JC model parameters. Numerical calculations of the Taylor rod-on-anvil impact test were performed for cylindrical copper specimens. The numerical simulation performed with the optimized JC model parameters was in good agreement with the experimental data received by the authors of this paper and with the literature data. The accuracy of simulation depends on the experimental data used. For all considered experiments, the calculation accuracy (solution quality) increased by 10%. This method, developed for selecting optimized material model constants, may be useful for other models, regardless of the numerical code used for high-velocity impact simulations.

## 1. Introduction

Numerical simulation of high-velocity interactions of deformable solids requires using material models that adequately describe material behavior at high strain-rates and temperatures. The role of shock-wave processes increases with an increase in the impact velocity. Known material models, such as the classical empirical Johnson–Cook (JC) model [1,2], that describe the change in yield strength (both hardening and softening) give different results compared to the experimental data with increasing plastic strain, plastic strain rate, and temperature. Classical material models developed for calculating the reliability of construction elements and structures at high impact velocities can give critically incorrect results. An adequate numerical description of the behavior of materials under dynamic loading leads to modifications in known empirical models, increasing the number of fitting parameters and therefore increasing the complexity of such models. Most researchers use commercial software products, in particular ANSYS/LS Dyna, that implement known material models with standard constants, causing difficulties with the adequate simulation of high-velocity shock-wave processes.

The JC constitutive model is widely used in many scientific and engineering applications. For example, Zhang C. et al. [3] used the JC model to simulate the deformation and fracture of fiber-reinforced polymer composites at normal and elevated temperatures. Xie H. et al. [4] calibrated the JC model parameters for melt-cast explosives under static and dynamic loading. The JC model has been applied to anisotropic materials, orthotropic aluminum alloy after modification and experimental selection of parameters [5], and anisotropic deformation analysis of high-strength 6XXX aluminum alloy sheets [6]. Sun X. et al. [7] combined the JC model with a fracture model for magnesium alloys, focusing on the choice of model parameters. Yang S. et al. [8] selected the parameters of the modified JC model for titanium alloy at elevated temperatures using a global optimization method based on the initial values received using a regression method. Yin W. et al. [9] used the JC model to estimate residual stress on the surface and within the blade of aircraft engines. The JC model has been actively applied to simulate the high-speed machining of titanium, aluminum, and steel samples [10,11,12,13,14]. However, the parameters of the JC model are determined experimentally at low strain-rates and, as will be shown below, may not accurately describe the high-velocity deformation of materials. The estimation of the model parameters will be discussed in more detail below. To perform such an estimation, we propose to use a set of classical Taylor tests together with numerical simulations.

The Taylor test is usually used to compare numerical and experimental results at high impact velocities and to estimate the dynamic characteristics of materials in the strain-rate range of 10^4^–10^5^ s^−1^. Originally, the Taylor test was designed to calculate the impact yield stress of a cylindrical specimen, including its residual length, after impact with a non-deformable target (rigid wall) using a simple model of a rigid plastic material [15]. This approach has often been used to determine the dynamic yield strength [16,17,18,19,20] and to select constitutive relations and constants in numerical simulations [21,22,23,24,25,26,27].

At present, experimental studies and numerical simulations of the Taylor test have been performed actively for a wide range of materials and loading conditions. Sen S. et al. [28] examined five different plasticity models, such as the JC model [1,2], the Zerilli–Armstrong model [29], the Steinberg–Cochran–Guinan–Lund model [30], the Mechanical Threshold Stress model, and the Preston–Tonks–Wallace model [31], and showed that the Taylor test can determine the parameters of the models with acceptable accuracy. Lee S. et al. [32] investigated strain hardening of austenitic stainless steel (AISI 304) over a wide range of strain rates (from quasistatic to 10^6^ s^−1^). Armstrong R.W. [33] presented the constitutive relations of metals, including α-titanium, copper, α-iron, and tantalum for a wide range of strain rates. The focus was on the Taylor high-velocity impact tests (solid cylinder) and the simulation of the strain characteristics. Gao C. et al. [34] developed a modified Taylor test using a split Hopkinson pressure bar and high-speed imaging to obtain stress–strain curves. The proposed method has been verified by experiments and finite element method simulations at different impact velocities. Jia B. et al. [35] used a single shear specimen to investigate the thermo-viscoplastic behavior of aluminum alloy (2024-T351) subjected to simple shear stress. A hybrid material model was developed and verified via numerical simulation of the Taylor test. Li J-C. et al. [36] performed the Taylor tests for projectiles with four types of nose shapes (blunt, hemispherical, truncated ogive, and truncated conical) and studied the characteristics of loading conditions depending on the nose shape and impact velocity. In [37], Li J-C. et al. applied the results of [36] to a numerical and experimental study of the high-velocity loading of a missile-borne recorder at different velocities using the Taylor impact test. Selyutina N.S. et al. [38] determined the dynamic yield strength of metals using the structural–temporal approach, but the range of interaction velocities was limited, which reduced the role of the shock waves. Pantalé O. et al. [39] simulated the dynamic tensile properties of materials using a specially designed target in the Taylor impact test to generate tensile deformation in its central area. Rodionov E.S. et al. [40] experimentally and numerically investigated the dynamic plasticity of oxygen-free high-conductivity copper (OFHC) at strain values of 0.3 and strain rates up to 1.7 × 10^4^ s^−1^. Experimental data for OFHC copper at higher impact velocities of 150–450 m/s are presented in [18,41].

Thus, articles devoted to the JC model and the Taylor test are published annually in the world scientific literature. Most of the articles report on the choice of material model parameters to ensure good agreement of numerical calculations with experimental data. This trend has no tendency to decrease.

This paper presents the numerical calculations of classical Taylor rod-on-anvil impact tests for cylindrical OFHC copper samples. A method for selecting the JC model constants using an optimization algorithm based on the Nesterov gradient-descent method [42] was proposed. To optimize the selection of the JC model constants, a solution quality function was proposed, which can estimate the deviation of the calculations from the experimental data and determine the optimal JC model parameters. The optimal JC model parameters were selected.

## 2. Formulation of the Problem

A 3D simulation of a cylindrical projectile impact with a non-deformable target (Taylor test) was performed. The material of the cylindrical sample (oxygen-free high-conductivity copper, OFHC) was chosen to compare the numerical results with the experimental data [41]. Simulations of the experiments [16,43] were conducted for copper specimens with corresponding initial conditions (geometric dimensions, velocity).

The 3D calculations were performed using an elastic–plastic medium model and our own research software (COMP3, v1.0) based on the modified finite element method developed by G.R. Johnson [44]. The system of basic equations and relations of the finite element method can be found in [45,46,47]. The COMP3 software has shown its adequacy for a wide range of high-velocity interactions of deformable bodies [48,49].

**Figure 1:**
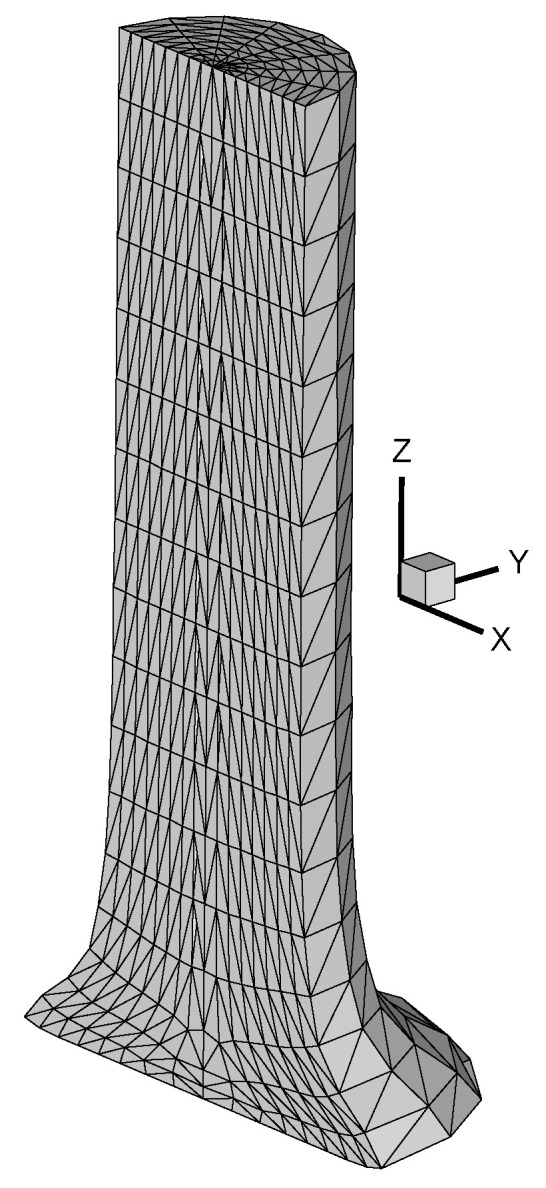
Finite element model of a cylinder. Diameter D_0_ = 7.8 mm, height L_0_ = 34.5 mm.

The method used 4-node tetrahedral finite elements. A finite-element mesh for a cylinder was generated as follows. The cylinder was divided along the height by planes parallel to the end surfaces into N_Z_—1 layers of the same height, where N_Z_ is the number of nodes along the vertical *Z* axis. Each layer was divided into N_Y_—1 equal sectors, where N_Y_ is the number of rays, and into N_R_—1 rings of equal thickness, where N_R_ is the number of nodes along the ray. As a result, the cylinder was divided into quadrangular and triangular (near the axis) prisms. The quadrangular prisms consisted of 6 tetrahedral elements, while the triangular prisms consisted of 3. Figure 1 shows the finite element model of a cylinder. This problem is symmetric, so 1/2 of the cylinder is modelled. The number of elements in the finite element model was 1314, and the number of nodes was 6120 (N_R_ = 9, N_Y_ = 9, N_Z_ = 18).

The material constants used in calculations were as follows: density (8930 kg/m^3^), bulk sound velocity (3940 m/s), shear modulus (41 GPa), yield strength (90 MPa), Grüneisen parameter (2.04), specific heat capacity (392.4 J/kg·K), Hugoniot adiabat coefficients (3940 m/s and 1.49) [16].

## 3. Modification of the JC Constitutive Model

The JC constitutive model [1,2] was used for the 3D simulation of deformation of a copper cylinder. The disadvantage of this model is that an increase in plastic deformation with an increase in the strain rate leads to an unlimited increase in the yield strength, which is not observed in experiments. In particular, Zelepugin S.A. et al. [41] showed that OFHC copper in high-velocity impact experiments with plastic strain accumulation demonstrates an increase in microhardness from 900 MPa to ~3000 MPa. Moreover, the JC constitutive model parameters were experimentally determined by the model developers at relatively low strain-rates (up to 10^3^ s^−1^). However, the strain rates reached values of 10^6^ s^−1^ and more at high-velocity impacts. Model constants determined for numerical simulation of high-velocity processes at low strain-rates led to discrepancies between numerical and experimental results. This raised the question of determining the parameters of the JC constitutive model for high strain-rates.

Numerical calculations using the original JC model at an impact velocity of 316 m/s revealed a hardening of ≥5 times due to an increase in equivalent plastic strains, which is 2 times higher than the hardening measured in [41]. In this regard, the JC constitutive model was modified by limiting the maximum value of plastic hardening in terms of the dimensionless factor ***B_max_***:

(1) $$\sigma =\min\left(\begin{array}{l} \sigma_{0}+B\varepsilon_{pl}^{n}\\ \sigma_{0}\cdot B_{\max} \end{array}\right)(1+C\ln({\dot{\varepsilon }}^{*}))\left(1-{\left(\frac{T-T_{0}}{T_{m}-T_{0}}\right)}^{m}\right)$$

Here, ***σ_0_*** is the quasi-static yield strength (denoted as ***A*** in the original model [1]); ***ε_pl_*** is the equivalent plastic strain; ${\dot{\varepsilon }}^{*}={\dot{\varepsilon }}_{pl}/{\dot{\varepsilon }}_{0}$ is the dimensionless plastic strain rate; ${\dot{\varepsilon }}_{pl}$ is the plastic strain rate; ${\dot{\varepsilon }}_{0}$ is the initial strain rate (${\dot{\varepsilon }}_{0}$ = 1 s^−1^); ***T_0_***, ***T***, and ***T_m_*** are the initial, current, and melting temperatures, respectively; and ***B***, ***n***, ***C***, and ***m*** are the material model constants.

## 4. Solution-Quality Function

We proposed to introduce a solution quality function to quantitatively estimate the discrepancy between experimental and numerical results.

**Figure 2:**
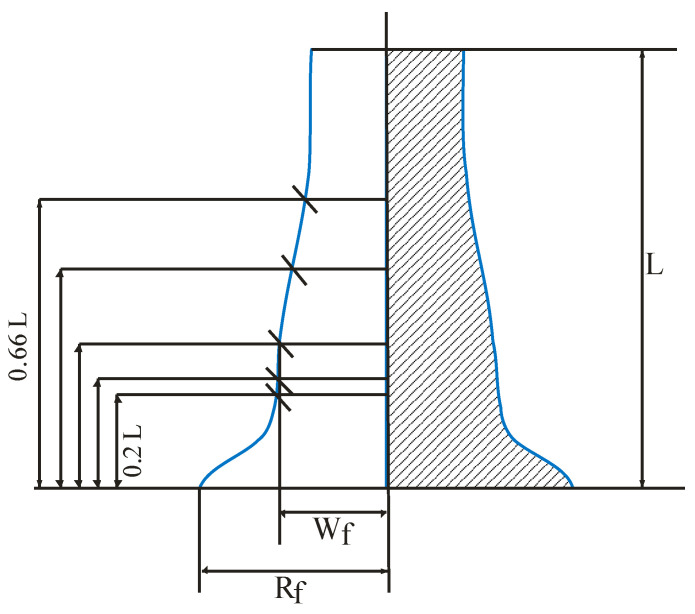
Cylinder measurement points after deformation.

The reliable measured parameters of the cylinder after impact are the residual length of the cylinder L and the radius of points on the cylinder lateral surface W_f_ (Figure 2). A less-reliable measured parameter is the maximum cylinder radius, R_f_, due to the possible fracture of the cylinder in contact with the rigid wall. It is worth noting that the specimens were partially broken along the external radius at impact velocities of 316 m/s and above [41].

For more accurate agreement between experimental data and numerical results, we proposed to estimate five points on the lateral surface of the cylinder. The solution quality function $Q_{f}$ was selected in the form as follows:

(2) $$Q_{f}=\left\{\begin{array}{c} 20\cdot \Delta {\left(\frac{L}{L_{0}}\right)}^{2}+\Delta {\left(R_{f}\right)}^{2}+\\ 2\left[\Delta W_{f}{\left(\frac{1}{5}\right)}^{2}+\Delta W_{f}{\left(\frac{1}{4}\right)}^{2}+\Delta W_{f}{\left(\frac{1}{3}\right)}^{2}+\Delta W_{f}{\left(\frac{1}{2}\right)}^{2}+\Delta W_{f}{\left(\frac{2}{3}\right)}^{2}\right] \end{array}\right\},$$

where

$$\Delta \left(\frac{L}{L_{0}}\right)=\left(\frac{L_{num}/L_{0}}{L_{exp}/L_{0}}\right)-1,\;\Delta W_{f}=W_{f}{}_{num}/W_{f}{}_{exp}-1,\;\Delta (R_{f})=R_{f}{}_{num}/R_{f}{}_{exp}-1.$$

The weighting factors 20, 2, and 1 were selected in such a way that the residual length, being a reliable measured parameter, had the greatest effect on the solution quality. Deviations of the lateral surface radius had less effect on the solution quality. The smallest contribution was given by the maximum radius, as it varied in the experiments during the tension of the external edge of the cylinder. Note that the quality function could be chosen in different ways, and the influence of quality function was not investigated in this paper.

The $Q_{f}$ function can numerically estimate the deviation of calculations from the experimental data and find the model parameters using optimization methods. $Q_{f}$ is a positive value and turns to zero when the calculations of the external surface of the cylinder after impact match the experimental data. The external surface of the cylinder remains smooth after deformation, so the chosen number and location of radius measuring points *W_f_* is adequate to approximate the shape of the external surface with high accuracy.

The introduced quality function $Q_{f}$ solves the problem of finding model parameters that minimize this function and are optimal.

Since the number of parameters to be optimized was small (6: ***σ_0_***, ***B***, ***n***, ***B_max_***, ***C***, ***m***), gradient-descent methods were effective for solving this problem. However, it should be noted that calculating the $Q_{f}$ value for a single numerical experiment takes a long time (up to several hours, Intel Xeon 3.2 GHz). Therefore, the number of calculation steps should be minimized. For this purpose, the Nesterov gradient-descent method was chosen to determine the $Q_{f}$ minimum [42].

The idea of the Nesterov gradient-descent method is that the direction of the gradient is calculated at the predicted value of the currently found point, and the movement is performed relative to this point. The convergence rate of the method increases if the gradient of the function changes lightly. In this case, the distance between the points grows, and the speed of movement along the gradient increases. At the same time, sharp changes in the gradient direction with error lead to a reduction in the movement step, and the method effectively finds the local minimum.

Numerical calculations showed that the influence of parameters m and n on the numerical results was weak for the considered impact conditions. In addition, the parameter σ_0_ is a quasi-static yield strength and cannot change at high strain-rates from a physical point of view. Thus, three parameters of the modified model remained under consideration: ***B***, ***B_max_***, and ***C***.

The JC model parameters were optimized to determine the minimum of the function:

(3) $$\begin{array}{l} \min\left[Q_{f}(\vec{x})\right]\\ \vec{x}=\left[B,B_{\max},C\right] \end{array}$$

## 5. Numerical Results and Discussion

The parameters of the Johnson–Cook model for OFHC copper were estimated using the experimental data of the Taylor test [41] and well-known experiments on the impact of cylinders with a rigid wall by Gust W.H. [16] and by Wilkins M.L. and Guinan M.W. [43]. An extended review of the calculated and experimental data for different medium models is given in [50].

For simulation, we chose experiments with the initial data shown in Table 1. The first series of calculations was performed using the original Johnson–Cook model parameters for copper, shown in Table 2.

**Figure 3:**
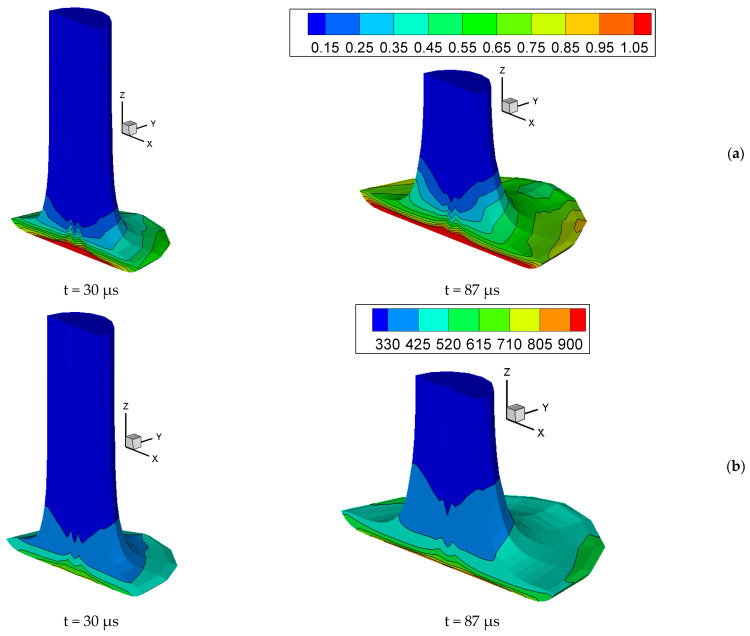
Fields and isolines of (**a**) specific shear strain energy (GJ/m^3^) and (**b**) temperature (K) in a copper cylinder at 30 and 87 µs upon impact with a rigid wall at an initial velocity of 316 m/s.

Figure 3a shows the fields and isolines of the specific shear strain energy, and Figure 3b shows the temperature fields and isolines at times of 30 and 87 µs. It can be seen that the maximum shear strains, as well as temperature, were generated in the center of the cylindrical specimen at the contact boundary between the specimen and the rigid wall. The maximum temperature reached 949 K by 87 µs. The calculations were terminated at 87 μs when the average velocity of the specimen along the *Z* axis became positive. We did not use a failure criterion and friction in our simulation [51].

**Figure 4:**
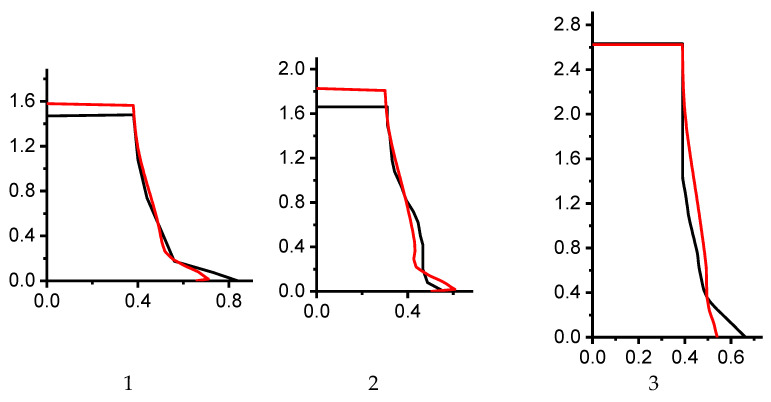

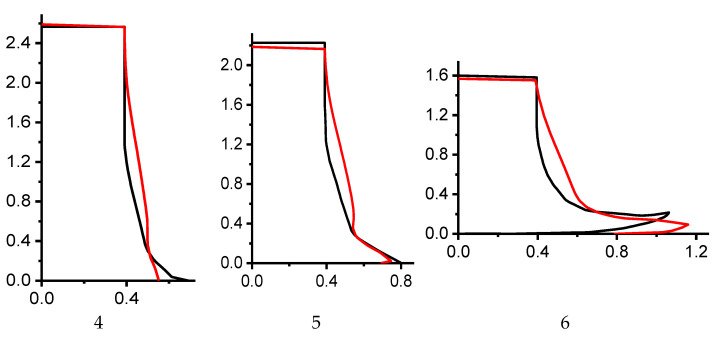
Calculated (red) and experimental (black) profiles of the external surfaces of the cylinders (original Johnson–Cook model parameters from Table 2); 1, 2, 3, 4, 5, 6 are the sequence number of tests in Table 1.

Calculated and experimental profiles of the external surfaces of the cylinders are shown in Figure 4.

The values of the solution quality function are presented in Table 3.

Calculations showed that the original JC model constants did not agree quantitatively with the experimental data. Tests 3 and 4 demonstrated a near-perfect match between numerical and experimental data for the cylinder’s final length. However, the shape of the lateral surface and the maximum diameter differed. For test 1, the calculation described well the experimental shape of the lateral surface, but there was a discrepancy in the cylinder’s final length. Other tests also showed a discrepancy between numerical and experimental results. It is worth noting that the simulation performed for tests 1 and 2 were in good agreement with the calculations presented in [50].

Optimization of the JC model parameters was performed as follows:

1. Optimization of parameters for each of tests 1, 2, 3, 4, 5, and 6;
2. Optimization of parameters for tests 1 and 2:

(4) $$Q_{f}=\frac{1}{2}\left(Q_{f}^{<1>}+Q_{f}^{<2>}\right);$$

3. Optimization of parameters for tests 3–6:

(5) $$Q_{f}=\frac{1}{4}\sum_{i=3}^{6}Q_{f}^{<i>};$$

4. Optimization of parameters for tests 1–6:

(6) $$Q_{f}=\frac{1}{6}\sum_{i=1}^{6}Q_{f}^{<i>}.$$

A universal code was developed to solve all four optimization problems simultaneously. The total number of Taylor test calculations exceeded 900. The solution time was about one day for an Intel Xeon 3.2 GHz.

**Figure 5:**
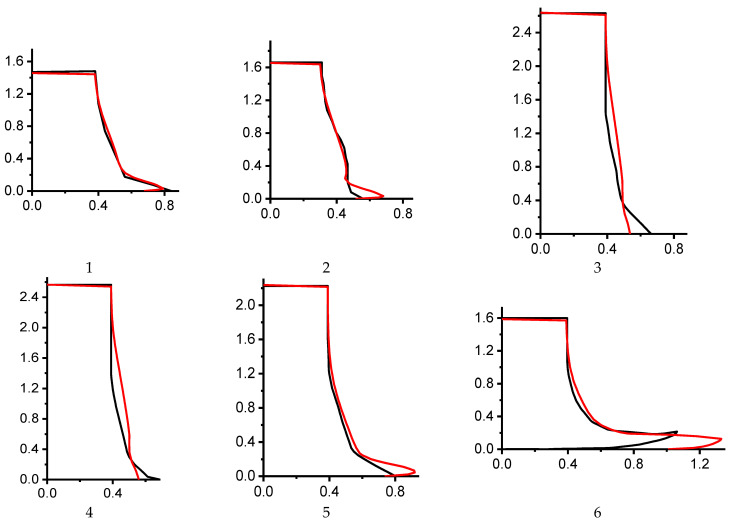
Calculated (red) and experimental (black) profiles of the external surface of the cylinders (optimal Johnson–Cook model parameters from Table 4) for tests 1–6.

Figure 5 shows the calculated and experimental profiles of the external surface of the cylinders when optimizing the parameters for each of experiments 1, 2, 3, 4, 5, and 6. The JC model parameters after optimization are presented in Table 4.

In tests 1–4, the hardening limit was reached in a small volume and did not affect the process. The material deformed heavily in tests 5 and 6, so the results were affected by the limitation of the maximum plastic hardening. For this reason, in tests 5 and 6, the numerical optimization reduced the hardening limit ***B_max_*** to a value of ~2.8, which corresponds to the microhardness experiments [41]. However, in the experiments [41], the increase in maximum microhardness at individual points reached 3.5 times, and the average microhardness increase at the front end of the cylinder increased by 1.8–2.3 times.

Table 5 compares the calculated optimal model parameters with the original parameters [2] for different tests, where ***B/B_0_*** and ***C/C_0_*** are the ratios between optimal and original parameters, *Q_f0_* is the solution quality before the optimization of parameters, and *Q_f_* is the solution quality after the optimization of parameters. Good agreement between the calculated and experimental data was observed at ***Q_f_*** < 0.07 (tests 1, 2, 5, 1 + 2, 3 + 4 + 5 + 6).

It should be noted that the original parameters of the Johnson–Cooke model better described the behavior of copper in the experiments in [41] at impact velocities of 162 m/s and 167 m/s than in the experiments in [16,43]. However, at higher impact velocities, the deviation of the optimal model parameters from those determined in the low-velocity experiments increased up to two times.

Calculations showed that the experiments in [16,43] are best described by the Johnson–Cooke model, assuming that the OFHC copper parameters differ from the measured parameters [2] (***B***-decrease by 30%, ***C***-decrease by 10%). At impact velocities of 162 m/s and 167 m/s [41], the experiments are described exactly by the same set of parameters. However, with an increase in the impact velocity, the deviation of the calculated values from the experimental values increases. The set of experimental data [41] is better described under the assumption that hardening parameter ***B*** of the used copper increases by a factor of 1.9, while parameter ***C*** decreases slightly.

Determining a set of parameters that optimally describes the set of experiments considered [16,41,43] showed that the average solution quality improves by 10%, but this improvement is achieved by the accurate description of the experiments in [16,43] (by 1.7 and 1.8 times) and a slight deviation (by 20% and 10%) in the description of the experiments in [41] for impact velocities of 225 m/s and 316 m/s, respectively.

## 6. Conclusions

1. The JC constitutive model was modified by introducing a material-hardening limit for plastic deformation, ***B_max_***, at high strain-rates.
2. A solution quality function, ***Q_f_***, was proposed to estimate the deviation of calculations from the experimental data. The final length of the cylinder, the radius of the lateral surface of the cylinder at five points, and the maximum radius of the cylinder were taken as the function parameters, with weighting factors of 20, 2, and 1 according to the effect on the final quality of the solution and reliability of the parameter measurement.
3. An optimization algorithm for selecting parameters ***B*** and ***C*** of the JC constitutive model and the limiter ***B_max_*** was developed to find the best agreement between the calculated and experimental data for the Taylor impact test using the Nesterov gradient-descent method.
4. The optimal parameters, namely, ***B***, ***B_max_***, and ***C***, of the modified JC constitutive model were calculated for nine sets of experimental data. The solution quality in some experiments increased by several times when using optimal parameters. For all experiments, the solution quality improved by 10% after optimization.
5. The developed method for optimizing the selection of the constitutive model constants can be adapted for a wide range of problems (arbitrary set of optimized parameters, arbitrary material models, and software codes, including ANSYS/LS Dyna).

## Figures and Tables

**Table 1:** Parameters of experiments.

Test	Material	L_0_ (mm)	D_0_ (mm)	ʋ_0_ (m/s)	T_0_ (K)	Reference
1	OFHC Cu	23.47	7.62	210	298	[43]
2	ETP Cu	30	6.0	188	718	[16]
3	OFHC Cu M1	34.5	7.8	162	298	[41]
4	OFHC Cu M1	34.5	7.8	167	298	[41]
5	OFHC Cu M1	34.5	7.8	225	298	[41]
6	OFHC Cu M1	34.5	7.8	316	298	[41]

**Table 2:** Original Johnson–Cooke model parameters [2].

*σ_0_* (MPa)	*B* (MPa)	*C*	*n*	*m*	*T_m_* (K)
89	292	0.025	0.31	1.09	1356

**Table 3:** Values of the solution quality function for tests 1–6.

Test	1	2	3	4	5	6	Average	Standard Deviation
$Q_{f}$	0.149	0.292	0.099	0.143	0.162	0.311	0.193	0.07

**Table 4:** Optimal parameters of the Johnson–Cook model.

Test	*B* (GPa)	*C*	*B_max_*
1	0.202	0.023	3.509
2	0.205	0.023	3.503
3	0.309	0.024	3.387
4	0.286	0.022	3.623
5	0.539	0.014	2.783
6	0.486	0.018	2.881
1 + 2	0.204	0.023	3.493
3 + 4 + 5 + 6	0.565	0.020	2.558
1 + 2 + 3 + 4 + 5 + 6	0.265	0.024	3.330

**Table 5:** Solution quality for optimal parameters of the Johnson–Cook model.

Test	*B/B_0_*	*C/C_0_*	*B_max_*	*Q_f_*	*Q_f0_*	*Q_f0_*/*Q_f_*
1	0.692	0.919	3.509	0.011	0.149	13.6
2	0.702	0.907	3.503	0.046	0.292	6.4
3	1.059	0.948	3.387	0.098	0.099	1.0
4	0.981	0.865	3.623	0.143	0.143	1.0
5	1.846	0.566	2.783	0.041	0.162	3.9
6	1.663	0.725	2.881	0.088	0.311	3.5
1 + 2	0.697	0.910	3.493	0.028	0.221	7.8
3 + 4 + 5 + 6	1.936	0.820	2.558	0.063	0.179	2.8
1 + 2 + 3 + 4 + 5 + 6	0.908	0.965	3.330	0.177	0.193	1.1

## Data Availability

Not applicable.
